# A Low-Profile Dielectric Resonator Filter with Wide Stopband for High Integration on PCB

**DOI:** 10.3390/mi14091803

**Published:** 2023-09-21

**Authors:** Shixian Lin, Mengdan Wang, Kai Xu, Lingyan Zhang

**Affiliations:** 1 School of Information Science and Technology, Nantong University, Nantong 226019, China; lsxheng@stmail.ntu.edu.cn (S.L.); dan107529@outlook.com (M.W.); xukaihopeness@hotmail.com (K.X.); 2 Research Center for Intelligent Information Technology, Nantong University, Nantong 226019, China; 3 Nantong Key Laboratory of Advanced Microwave Technology, Nantong University, Nantong 226019, China

**Keywords:** bandpass filter, dielectric resonator, high integrated, printed circuit board (PCB) process

## Abstract

A low-profile dielectric resonator (DR) filter is proposed to achieve the feature of high integration and wide stopband. The high integration is due to the structure of printed circuit board (PCB) substrate instead of metal cavity, which can be easily integrated with other planar circuits. Thus, the proposed design can improve the integration level and reduce installation errors. Moreover, the out-of-band harmonics can be well suppressed by the structure combined with introducing rectangular hollowing in the center of the dielectric block, coupling the feed and loading 1/4λ wavelength branch. For demonstration, it is fabricated and measured. The simulated and experimental results with good agreement are presented, the insertion loss is as low as 1.1 dB, the profile height is only 0.77*λ*_g_, and the stopband reaches 2.61*f*_0_.

## 1. Introduction

Dielectric resonator (DR) [1,2,3,4,5,6,7] filters have become a research hotspot in recent years due to their superior characteristics such as high Q value, low manufacturing cost, temperature stability, miniaturization, and compatibility with microwave circuits. The wide stopband filter [8,9,10,11,12] has also been widely used in microwave systems because of its good harmonic suppression level, which can meet the out-of-band requirements.

With the rapid growth of communication, various approaches have been reported to achieve wide stopband DR filters. The first method is to use the balanced structure of the dielectric resonator filter to suppress harmonics [13,14], because differential feeding itself is a mode selection, but the differential feeding will produce a common mode response, and the early appearance of common mode is the main reason for inhibiting wide stopband. The second method [15,16,17] is to combine two different modes of dielectric resonators, which can significantly improve the spurious performance. However, due to the combination of two different types of resonators, the filter size is too large and the structure is complex. In order to avoid the above problems, the third method [18,19,20,21] starts from the resonator itself and introduces a cylindrical hole in the center of the dielectric resonator to improve the separation between the dominant mode and the higher-order mode, but the stopband bandwidth expansion is limited.

**Figure 1:**
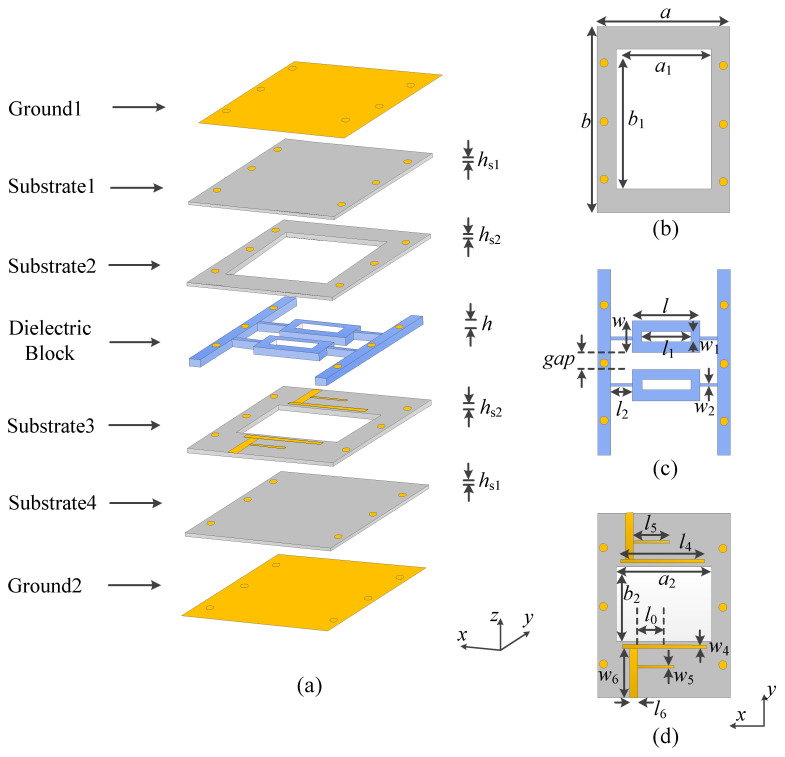
The structure of the proposed filter. (**a**) Three-dimensional view of the proposed DR filter. (**b**) Substrate 2. (**c**) Dielectric block. (**d**) Substrate 3.

In this letter, a low-profile DR filter with a wide stopband as shown in Figure 1 is proposed, which uses a substrate instead of the traditional metal cavity structure to reduce the profile height and improve the integration. Benefiting from the structure of the coupled feeding, not only are some unnecessary modes filtered, but also the direct contact between the feed structure and the dielectric block is avoided, and the assembly error is reduced. More importantly, the low frequency harmonic mode of the dielectric resonator is suppressed by digging a rectangular groove in the center of the dielectric block, and the high frequency harmonic mode is suppressed by the structure of loading branches. Finally, the excellent suppression level of the filter is achieved. For demonstration, a DR filter with a wide stopband is designed and fabricated.

## 2. Proposed Low-Profile Dielectric Resonator Filter with Wide Stopband

### 2.1. Methodology and DR Filter Design

Figure 1 exhibits the structure composition of the proposed low-profile dielectric resonator with a wide stopband. The top and bottom are metal layers, with four substrate layers and a dielectric layer stacked in the middle. Substrates 1, 2, 3 and 4 are Rogers 4003C (dielectric constant *ε_r_* = 3.38 and loss tangent of 0.0027) with thicknesses of 0.203, 0.813, 0.203, and 0.813 mm, respectively. Substrates 2 and 3 are hollowed with air cavities to reduce the dielectric loss and enhance the quality factor. The air cavity of substrate 3 is smaller than substrate 2, because metal feeders are printed on substrate 3. Substrates 1 and 4 are both complete substrates, which are used to print the complete metal ground. The media block layer consists of two main rectangular dielectric blocks, four connecting strips and fixed strips on both sides, with dielectric constant *ε_r_* = 89.5 and a loss tangent of 0.0006.

The input/output coupled lines and the feed lines are implemented on substrate 3 as shown in Figure 1d, allowing for transmission between signals. The top and bottom metal layers of the substrate are connected through metal vias, so that the whole circuit can be packaged. Each dielectric block has a rectangular hollow in the center, which can suppress harmonics. The software for full-wave simulation is Computer Simulation Technology (CST).

**Figure 2:**
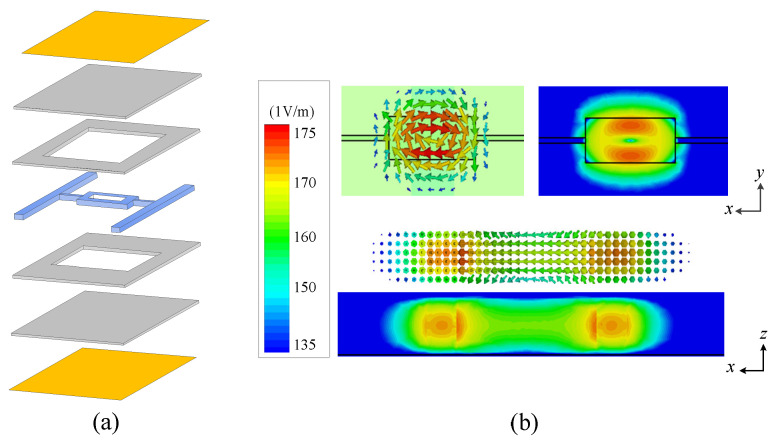
(**a**) The simplified model of the proposed HIDR. (**b**) The electric field of ${TE}_{11\delta }^{z}$ mode.

The simplified model in Figure 2a is used to analyze the proposed DR, where the dielectric block layer consists of a rectangular block (*a* × *b* × *h*), two connecting strips and two fixed strips with two layers of dielectric substrate and metal layer on the top and bottom, respectively. Figure 2b shows the working mode (${TE}_{11\delta }^{z}$ mode) of the proposed DR. It can be seen that the strong electric field of the mode is concentrated above and below the center symmetry, and it is distributed in a circular pattern. For the ${TE}_{11\delta }^{z}$ mode, there is no electric field passing through the *z* direction, thus it can be named as TEz mode. Additionally, 1, 1 and δ mean the number of half-wavelength distribution, where 0 < δ < 1.

According to Helmholtz equation and boundary conditions, the EM field expressions of ${TE}_{11\delta }^{z}$ mode can be presented as follows:

(1) $$k_{x}=\frac{m\pi }{l},k_{y}=\frac{n\pi }{w}$$

(2) $$\varepsilon_{reff}=[(\varepsilon_{r1}\times t)+(\varepsilon_{r2}\times h)]/(t+h)$$

(3) $$h_{reff}=h+t$$

(4) $$k_{x}^{2}+k_{y}^{2}-\alpha_{z}^{2}=k_{0}^{2}$$

(5) $$k_{x}^{2}+\alpha_{z}^{2}=(\varepsilon_{reff}-1)k_{0}^{2}$$

(6) $$f_{0}=\frac{c}{2\pi \sqrt{\varepsilon_{reff}}}\sqrt{k_{x}^{2}+k_{y}^{2}+k_{z}^{2}}$$

where *ε_reff_* is the effective dielectric constant, *h_reff_* is the effective height, *k*_0_ is the wavenumber at operation frequency, *ε_r_* is the relative permittivity of the DR, and *k_x_*, *k_y_*, *k_z_* are the wavenumber in the three directions of *x*, *y*, *z*.

**Figure 3:**
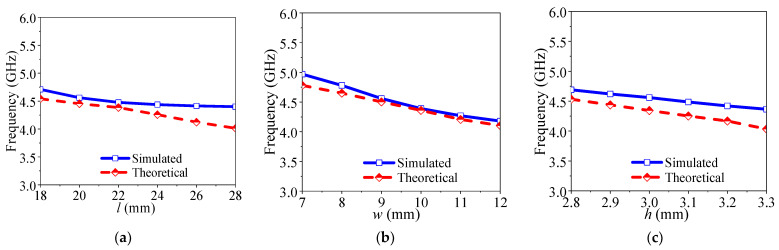
The variations of resonant frequency for ${TE}_{11\delta }^{z}$ mode of the DR when changing *a*, *b* and *h*. (**a**) Different *a*. (**b**) Different *b.* (**c**) Different *h*.

Figure 3 exhibits how the variation of resonant simulation frequency and theoretical frequency of ${TE}_{11\delta }^{z}$ mode in DR vary with *a*, *b* and *h*. It can be seen that the resonant frequencies of simulation and theoretical values of ${TE}_{11\delta }^{z}$ mode are in good agreement. It can be found that with the increase in *a*, *b* and *h*, the simulation and theoretical frequencies are decreasing.

**Figure 4:**
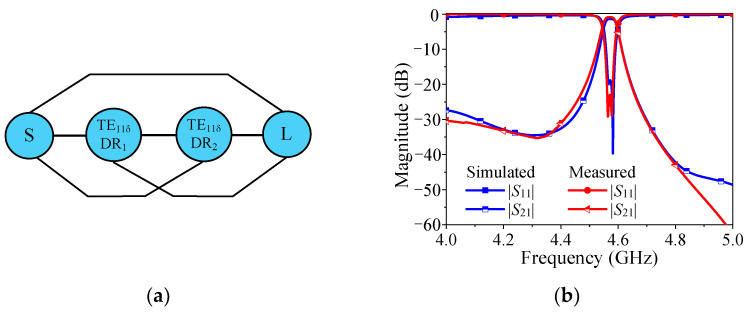
The coupling topology and the theoretical response of the proposed design. (**a**) Coupling topology. (**b**) Theoretical and simulated response.

The coupling topology of the proposed DR filter with wide stopband is given in Figure 4, where the *S* and *L* represent the source and the load, respectively. Owing to its symmetric configuration, the coupling factors satisfy the relationship of *M*_S1_ = *M*_2L_, and *M*_S2_ = *M*_2L_, where *M* is the coupling between different elements in the coupling matrix. Here, 1 and 2 represent the first resonator and the second resonator, respectively.

For demonstration, a low-profile DR filter with wide stopband is designed. We assume that the order n is equal to 2. According to the proposed filter design index, we can calculate the lumped element values in the low-pass prototype filter, and the results are as follows: *g*_0_ = l, *g*_1_ = 0.8431 and *g*_2_ = 0.6220 [22]. Therefore, the coupling coefficient *k*_12_ and external quality factor *Q_e_* required by the filter design can be calculated by the following formula [22]:

(7) $$k_{1^{A}1^{B}}=\frac{FBW_{L}}{\sqrt{g_{1}g_{2}}}=0.00713$$

(8) $$Q_{e1}=\frac{g_{1}g_{2}}{FBW_{L}}=163.27$$

Assuming filter with the center frequency of *f*_0_ = 4.97 GHz and the 3-dB fractional bandwidth (FBW) of 0.99%, the coupling matrix *M* can be written as

(9) $$M=\left[\begin{array}{ccccc} & S & 1 & 2 & L\\ S & 0 & 0.8163 & 0.0151 & 0.0008\\ 1 & 0.8163 & 0 & 0.7625 & 0.0151\\ 2 & 0.0151 & 0.7625 & 0 & 0.8163\\ L & 0.0008 & 0.0151 & 0.8163 & 0 \end{array}\right]$$

Figure 4b shows the theoretical response resulting from the coupling matrix, it can be found that the theoretical response matches well with the simulated one.

**Figure 5:**
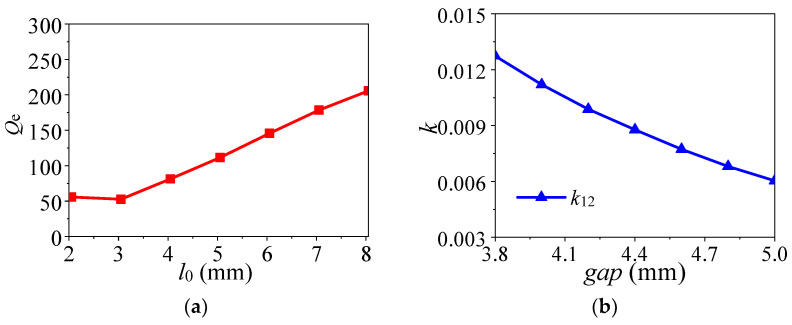
Extracted *Q_e_* and coupling coefficient. (**a**) *Q_e_* against *l*_0_. (**b**) *k*_12_ against *gap*.

The required external quality factor and coupling coefficients can be calculated from the lowpass prototype parameters. As a result, the desired *Q_e_* = 163.27 and *k*_12_ = 0.00713 are obtained, which are used for determining the main coupling. In this design, *Q_e_* is mainly controlled by the position of the tapping point *l*_0_, while *k*_12_ is determined by the coupling space *gap*, as shown in Figure 5a and Figure 5b, respectively.

### 2.2. Parametric Study and Performance Analysis

**Figure 6:**
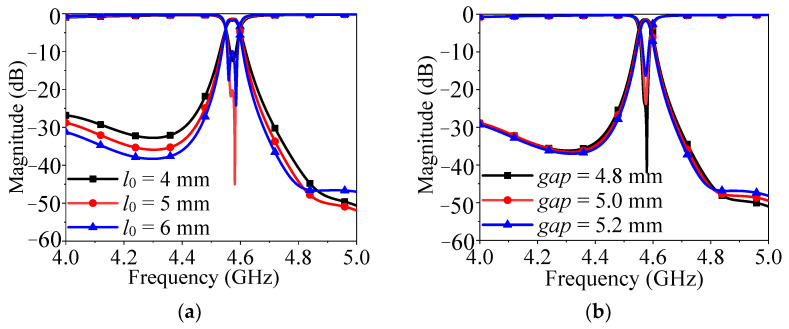
Effect of different parameters on simulated performance of the proposed design. (**a**) Different *l*_0_. (**b**) Different *gap*.

To clear the performance variation of the proposed design, Figure 6 exhibits the effects of different parameters (*l*_0_ and *gap*). Figure 6a shows that the impedance matching changes with the change of *l*_0_, because the feeding position affects the external quality factor. As can be seen from Figure 6b, as the gap increases, the coupling between the two DRS becomes weaker and the bandwidth decreases. In addition, the impedance matching first becomes better and then worse.

**Figure 7:**
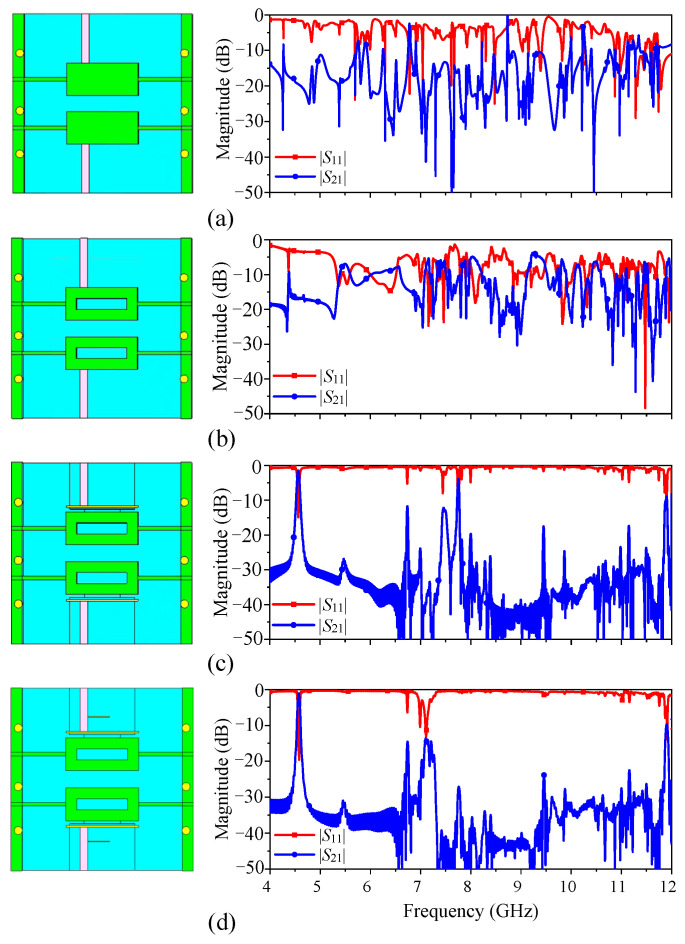
Filter structure changes and performance response. (**a**) Full resonator combined with direct feed. (**b**) Loaded rectangular slot resonator combined with direct feed. (**c**) Loaded rectangular slot resonator combined with coupled feed. (**d**) Loaded rectangular slot resonator combined with coupled feed and 1/4 wavelength branch.

In order to meet the requirements of the filter on the out-of-band harmonic suppression level, this design adopts the structure of the combination of three methods. As shown in Figure 7a, the structure is realized by using the resonator without loading rectangular slots and directly feeding. It can be seen from the corresponding response diagram on the right that there are more harmonics in the low and high frequency bands of the structure, and the out-of-band response is poor. The structure shown in Figure 7b is realized by loading the rectangular slot resonator combined with direct feeding. It can be seen from the response diagram that the structure effectively inhibits some harmonics at the low frequency band. By introducing rectangular hollowing in the center of the dielectric block, the modes with strong electric field distributions in the center of the dielectric block can be suppressed effectively. It can be seen from Figure 7a,b that a mode (TEy 111 mode) located at 5 GHz is suppressed by hollowing the rectangular slot in the center of the dielectric block.

The structure shown in Figure 7c is realized by loading a rectangular slot resonator combined with coupling feeding. Compared with direct feeding, the structure can suppress multiple harmonics in the band, and the coupling feed will not let the feeder make contact with the medium block directly, which is helpful to reduce the subsequent assembly error. Finally, Figure 7d shows the structure of loading a 1/4 wavelength branch, which can effectively suppress harmonics in high frequency bands. Therefore, the structure combined with these three methods can effectively solve the problem of poor out-of-band performance of the filter and improve the stopband performance.

### 2.3. Design Procedure

The design procedure of the proposed DR filter can be simply summarized as follows:

(i) Obtain the dimensions of the high integrated dielectric resonators according to the desired center frequency from the resonant mode frequency variation.
(ii) Achieve *l*_0_ according to the coupling matrix and impedance matching parametric study in Figure 6a.
(iii) Obtain *gap* according to the coupling matrix and bandwidth parametric study in Figure 6b.
(iv) Optimize the performance and obtain the final dimensions in CST.

## 3. Results

**Figure 8:**
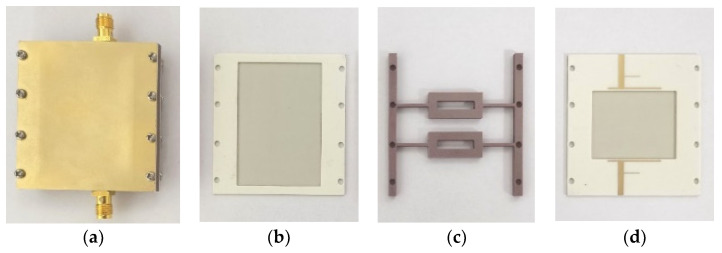
Photograph of the prototype. (**a**). Integral package diagram. (**b**) Substrate 2. (**c**) Dielectric block. (**d**) Substrate 3.

A prototype of the proposed low-profile DR filter is demonstrated. The detailed dimensions are shown as follows: *a* = 50 mm, *b* = 50 mm, *a*_1_ = 4 mm, *b*_1_ = 40 mm, *a*_2_ = 35 mm, *b*_2_ = 30 mm, *l* = 18 mm, *w* = 9 mm, *l*_1_ = 14 mm, *w*_1_ = 3 mm, *l*_2_ = 12 mm, *w*_2_ = 1 mm, *w*_3_ = 2 mm, *l*_4_ = 18 mm, *w*_4_ = 0.5 mm, *l*_5_ = 7 mm, *w*_5_ = 0.3 mm, *l*_6_ = 7 m, *w*_6_ = 1.8 mm, *h* = 3.1 mm, *h*_s1_ = 0.203 mm, *h*_s2_ = 0.813 mm. Figure 8 shows photos of the prototype, simulations, and measurements by the Keysight N5230C vector network analyzer (Santa Rosa, CA, USA). Among them, Substrates 1 and 2 (Substrates 3 and 4) are integrated and processed together during the fabrication processing through one prepreg layer with the thickness of 0.1 mm, dielectric constant of 3.38 and loss tangent of 0.0027, while the dielectric block is fabricated separately. Then, assemble them together by using metal screws. The limitation of this kind of assembly method is composed of two parts. Firstly, the tightening of assembly metal screws cannot be guaranteed, which will reduce the unloaded quality factor of the dielectric resonator and thus increase the loss of the filter. Secondly, the deviation in the relative position of the substrates and the dielectric block cannot be avoided, thus the impedance matching can be influenced and changes the bandwidth accordingly.

**Figure 9:**
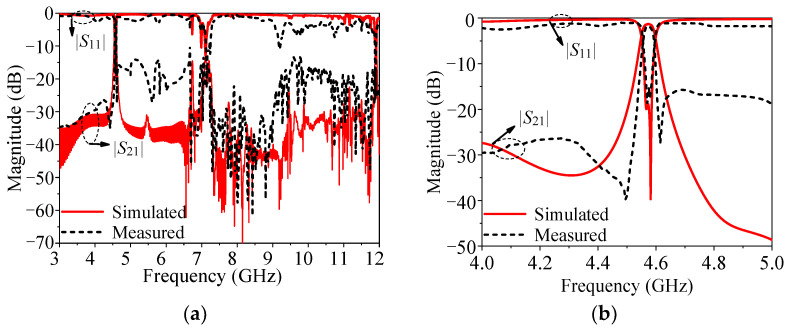
Simulated and measured results of the prototype. (**a**) Full band response. (**b**) Enlarged response.

Figure 9 exhibits that the simulated minimum insertion loss is about 1.23 dB at 4.57 GHz, the simulated 3-dB bandwidth is about 1.05%, and the simulated harmonic suppression is about 2.62*f*_0_. The measured minimum insertion loss is about 1.29 dB with the center frequency of 4.57 GHz, the measured 3-dB FBW of 0.99% and the harmonic suppression reaches 2.61*f*_0_. The deviation between the simulated and measured results is due to the fabricated error, assembly error and the measured error.

Table 1 lists the performance and state-of-the-art design of this work. Compared with existing dielectric resonator filters with wide stopbands, this design improves integration, reduces the profile, and improves the harmonic suppression level. Here, the improved integration is attributed to the PCB technology, which can be integrated with other planar circuits that were fabricated on the PCB.

## 4. Conclusions

A low-profile DR filter with wide stopband is proposed. By using the grounded substrate to replace the traditional metal cavity, the proposed one shows high integration with other planar circuits. Moreover, it benefited from rectangular hollowing in the center of the dielectric block, coupling feed and loading 1/4λ wavelength branch, it obtains a wider stopband than other DR filters. A prototype with high integration is fabricated at 4.57 GHz, and obtains the profile of 0.77*λ*_g_ and a wide stopband up to 2.61*f*_0_. Therefore, the proposed DR filter can improve the integration by sharing the same substrate with other circuits, reduce the cost, and improve the level of out-of-band harmonic suppression. It is believed that this design has broad application prospects in modern wireless communication systems.

## Figures and Tables

**Table 1:** The proposed antenna compared to other related works.

Ref. No.	*f*_0_ (GHz)	3-dB FBW (%)	Insertion Loss (dB)	Q_u_	Profile (*λ*_g_)	Wide Stopband	Easy to Integrate
[11]	1.82	0.32	0.55	N.A	1.12	1.22*f*_0_	No
[12]	3.967	5	1.28	1077	0.985	1.05*f*_0_	No
[13]	1.755	N.A	0.5	5000	1.26	2.5*f*_0_	No
[14]	1.75	1.34	0.8	N.A	1.19	2.12*f*_0_	No
[15]	2.014	1.19M	N.A	2550	2.78	>15 dB up to 2.135*f*_0_	No
[16]	5.9	0.35	1.3	5500	N.A	1.7*f*_0_	No
[17]	5.15	55	0.32	N.A.	N.A	1.45*f*_0_	No
[18]	4.0	40	0.25	5000	0.704	1.15*f*_0_	No
[19]	1.86	20	0.5	N.A	1.57	1.24*f*_0_	No
This work	4.57	0.99	1.29	1315	0.77	>13.6 dB up to 2.61*f*_0_	Yes

*λ*_g_: The guide wavelength at the center frequency.

## Data Availability

The data presented in this study are available on request from the corresponding author.
